# Exploring the effects of health information seeking on e-satisfaction in online health communities: an empirical investigation

**DOI:** 10.1186/s12911-022-02079-y

**Published:** 2022-12-16

**Authors:** Pei Wu, Runtong Zhang

**Affiliations:** Department of Information Management, School of Economics and Management, Beijing Jiaotong University, Beijing, China

**Keywords:** Health information seeking, E-satisfaction, Social information processing theory, Social exchange theory, Structural equation model

## Abstract

**Background:**

Online health communities (OHCs) are becoming effective platforms for people to seek health information. Existing studies divide health information into general and specific information in OHCs. However, few studies discuss the effects of different types of information seeking in OHCs on users’ electronic satisfaction (e-satisfaction).

**Objective:**

This study explores the effects of general and specific information seeking on users’ e-satisfaction with OHCs through the mediating roles of perceived benefits and costs drawing on the social information processing theory and the social exchange theory.

**Methods:**

This study conducted an online survey to collected data from individuals who used OHCs to seek information. The structural equation model was used to analyze the collect data and the research model. Specifically, this study examined the common method bias and conducted a robustness check.

**Results:**

Results show that general and specific information seeking affect e-satisfaction through the mediating roles of perceived benefits and costs. An interesting result is that general information seeking has a stronger effect on e-satisfaction than specific information seeking.

**Conclusions:**

This study suggests that e-satisfaction should be further enhanced by information seeking as online healthcare practices evolve and change. Managers of OHCs should focus on increasing users’ perceived benefits, thereby increasing their e-satisfaction. Besides, this study discusses implications, limitations, and future research directions.

## Introduction

With the rapid development of information and communication technology (ICT), the way people seek health information has been enriched [1, 2]. Using the ICT to seek information increases users’ empowerment in health decision-making and promotes users’ communications with health professionals [3]. Online communities provide users with a piece of broad information on medical conditions, prevention strategies, and treatment options [4–6]. Online health communities (OHCs) represent one of the fastest-growing online communities and plausibly provide communications beyond the restrictions of geographic proximity [1]. Data from the Pew Research Center show that 72% of adults have used the Internet to search online about various health issues, the most popular of which concern specific diseases and treatments [7, 8].

Due to dissatisfaction with the information provided by offline health services, people visit online platforms to seek information on reasonable opinions from others [9–11]. Online platforms break the time and location constraints of the conventional information seeking processes, improving access to health information for marginalized populations and those living in remote areas [12, 13]. Online platforms are attractive to users because of convenience, anonymity, and diversity of information [14]. Users participate in OHCs partly to meet information seeking and acquire social support [15].

OHCs have the potential to increase the availability of health information more equally [16]. More than 90% of patients search for medical and therapeutic experiences similar to their diseases [15]. OHCs are an information hub where people can access every bit of information available about a topic [17]. Users can seek information on health risk assessments, health care recommendations, and similar illness experiences in OHCs [18]. An advantage of OHCs is the availability of information to meet users’ personalized information seeking anytime and anywhere [19]. Patients can get more convenient and high-quality medical services in OHCs. For example, previous studies have found that patients with emotional abnormalities who use online communities have perceived benefits from health information seeking [20].

Users can better communicate with health professionals, understand symptoms, and improve their understanding of treatments and side effects [21]. In the context of OHCs, users typically consider online interactions as advantageous for health outcomes, management of personal health conditions, relationships with physicians, and utilization of online health services [22, 23].

Health information seeking in OHCs can be classified as general and specific information seeking according to whether health information is specific to patients [24, 25]. General information is publicly available [26], defined as public health information on online communities, such as hospital information and pharmaceutical prices [24]. Publicly available information is developed from credible scientific and institutional sources [15]. General information is considered to be more likely credible information because it comes from a trustworthy source [27]. In addition, general information in OHCs can help users who are unsure what information they need. Specific information is related to personal experiences and physical illnesses [15]. Due to user-generated content, specific information has little source credibility, accuracy, currency, and completeness [24].

However, users always perceive information overload because of the extensive information in OHCs [28]. Health information seeking imposes users’ perceived costs in OHCs. Users are surrounded by misleading and confusing information [29]. Professionals provide clinical expertise and reliable information for individuals’ inquiries in OHCs. However, users nevertheless continue to rely on unverified information that comes from various sources [23]. Online information seeking requires users to understand the information of e-health literacy, computer skills, and institutional policies [13, 30, 31], which consumes lots of time and energy.

In addition, health professionals report several negative effects of participating in OHCs, including feeling overworked, overcommitted, insecure, and helpless in both their personal and professional lives [32]. Potential drawbacks of relying on the Internet for health-related information include information overload, navigational challenges, and quality issues [16]. Moreover, users are intimidated by or unaware of how to use operational technology [33]. In the process of health information seeking, users in OHCs are perceived costs due to these negative effects. However, few studies examine how users’ information-seeking affect their perceived benefits and costs. To narrow the research gap, this study focuses on users’ perceived benefits and costs in general and specific information seeking in OHCs.

E-satisfaction (electronic satisfaction) refers to people’s satisfaction with previous purchase experience in a certain electronic commerce (e-commerce) company [34]. E-satisfaction is a measurement of how satisfied users are with OHCs and the informational content. Health information seeking in OHCs saves unnecessary time in a one-to-one consultant and may benefit for physicians’ follow-up communication with patients. Previous studies have explored customers’ e-satisfaction and identified the positive effect of performance expectations and e-satisfaction [35]. Convenience motivation, perceived value, and trust significantly accentuate the relationship between e-satisfaction and e-loyalty [34].

Although e-satisfaction is getting scholars’ attention [35], few research explore users’ e-satisfaction with OHCs. To address the research gap, we examine the effects of users’ general and specific information seeking on their e-satisfaction with OHCs through the mediating roles of perceived benefits and costs. Based on the above arguments, we strive to answer the following research questions:

RQ1. How does general and specific information seeking affect e-satisfaction through the mediating roles of perceived benefits and costs in OHCs?

RQ2. How does general and specific information seeking affect perceived benefits and costs?

This study makes several contributions to health informatics and online platforms research. First, this study enriches the literature on users’ general and specific information-seeking in OHCs. This study examines the effects of general and specific information seeking on users’ e-satisfaction with OHC. The effective channel to users’ perceived costs and benefits is information seeking, which also has an impact on how satisfied they are with OHCs. Second, this study extends the understanding of the social information process theory in the context of OHCs. By investigating users’ perceptions of information seeking, this study reveals the benefits and costs of information processing in OHCs. This study empirically examines the mediating effects of perceived benefits and costs on the relationships between different types of information-seeking and e-satisfaction. In practice, this study provides implications for marketing OHCs to fill the health information gap in China.

## Literature review and theoretical background

Due to the value of ICT in healthcare, research on information seeking in OHCs has been growing exponentially. For example, acquiring the perceived usefulness of health information [36, 37], reducing the costs of acquiring health information [38], and assisting patients’ consultation decisions [39]. However, literature exploring the effects of different information-seeking on users’ e-satisfaction is finite in China. The success of OHCs relies on users’ participation in activities. The quality and quantity of information are significant for information-seeking in OHCs [40]. Few studies discuss how to increasing users’ e-satisfaction through general and specific information seeking. Based on the social information processing theory and the social exchange theory, this study investigates the mediating roles of perceived benefits and costs on the relationships between information seeking and e-satisfaction with OHCs in China.

### Social information processing theory

Social information processing theory suggests that people adapt their attitudes and behaviors to environments [41]. Social environments draws attention to information on individuals’ activities and attitudes [42]. people rely on social information to shape their attitudes, beliefs, and views when they have inadequate information about the work environment [43]. Based on the social information processing theory, people evaluate situations using social information [44, 45] People can derive maximum benefit and minimize personal risk after rationally assessing the appropriateness of information and behaviors [46, 47].

In addition, social information processing theory assumes that the nature and content of information may vary depending on social context [43]. Based on the social information processing theory, scholars have studied various of workplace phenomena, including employee job satisfaction and adaptation to new technologies [48, 49]. As an emerging social environment, OHCs provide users with the way to seek health information and adapt their attitudes.

Users evaluate general and specific information seeking perceived benefits and costs according to the social information processing theory. These psychological perceptions make users have favorable attitudes toward information-seeking in OHCs. Previous research has considered users’ satisfaction as an attitude [50]. Social information processing theory provides an essential perspective for studying different information seeking and the effects of psychological perceptions on e-satisfaction with OHCs.

### Health information types

ICT is changing the way that people gain information on health and disease [51]. The intersection of healthcare and online platforms provides enormous potential for facilitating online health services [52]. Online health information refers to health services information on online platforms, which strengthens users’ participation in health maintenance and treatment decisions [53–55]. Existing studies believe that health information is a relatively broad category, including disease information, health, physical and mental health, and other content [24]. Information is divided into two categories according to whether it is unique to those patients or not: general and specific information [24].

General information includes hospitals’ records, health services prices, and physicians’ public information in OHCs. In contrast, specific information includes patients with online reviews, the emotions of online consultations, and health outcomes. Moreover, general information is exceptionally rich in OHCs, which is determined valid information [56]. However, compared to general information, specific information involves users’ health issues. Previous studies have classified the information needs of informal professionals and information needs in general [57]. In this study, we classify health information in OHCs into general and specific information based on different users’ information needs. Specific information seeking is related to patients’ demands in OHCs. General information seeking in OHCs helps users who are not sure what information they need.

### Perceived benefits and costs

Social exchange theory proposes that individuals weigh benefits and costs through social behaviors before deciding whether to continue them [58]. Social exchange theory assumes that behaviors depend on maximizing benefits and minimizing costs [59]. Prior studies have discussed information seeking based on the social exchange theory and proposed that perceived benefits motivate users’ reciprocated intentions of information seeking [60]. There are advantages to taking part in online communities. The literature argues that users will contribute information in online platforms if there are benefits, such as rewards, reputation, reciprocity, and enjoyment [61, 62].

Users in OHCs connect with others who share their health issues [63]. People join OHCs primarily for health information and emotional support, such as friendship, encouragement, and sympathy [64]. Previous studies suggest that users’ perceived benefits from engaging in OHCs consist of utilitarian benefits1, symbolic benefits [24], and intrinsic benefits [65]. Perceived benefits come from non-instrumental, experiential, emotional, and personally gratifying benefits [66]. When users conduct online consultations, they are likely to make positively respond to health decisions [67]. Users’ information seeking in OHCs brings benefits in the processes of online consultations, such as information convenience, anonymity, and diversity of information sources [14]. Patients with emotional abnormalities who use online platforms perceive benefits [20]. Seeking behavior mainly promotes physician–patient interactions in clinical treatment, which is the embodiment of positive physician–patient relationships [68].

In contrast, costs refer to the expenditure of behaviors. When a potential detriment or cost is perceived, people are selfish to give up activities [69]. Prior studies suggest information exchanging makes users lose authority [70, 71]. Health professionals reject using OHCs as information seeking on online platforms requires them to spend non-chargeable hours and give up personal energy [36]. In addition, users recall their memory of experiences when seeking specific information that leads to painful and uncomfortable feelings. Users must cognitively identify the correctness and credibility of information in OHCs. Information exchange in online platforms requires the explanation and codification of information, which entails costs like time and labor [72, 73]. Information seeking in OHCs causes perceived costs for both physicians and patients.

## Research model and hypotheses

**Fig. 1:**
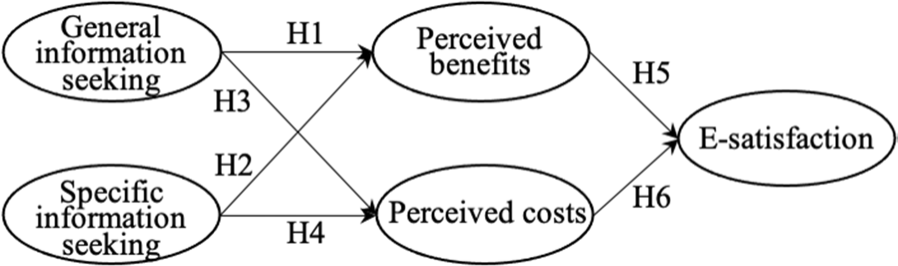
Research model

Based on the social information processing theory and the social exchange theory, we propose the research model (Fig. 1) to explore the effects of information seeking on e-satisfaction. Our research model includes two independent variables (general information seeking and specific information seeking), two mediation variables (perceived benefits and perceived costs), and one dependent variable (e-satisfaction). Users’ perceived benefits and costs are caused by general and specific information-seeking process. Users’ e-satisfaction with OHCs is affected by their perceived benefits and costs.

### Effects of general and specific information seeking on perceived benefits

ICT has increased access to general and specific information for people who are in remote areas and have inadequate health information [1]. The time and space barriers of the traditional information-seeking process are broken by ICT [74]. Users acquire perceived benefits from general information-seeking in OHCs, such as information support, and emotional companionship [75]. General information is publicly available and easily accessible. People are willing to obtain useful and reliable health information to make better medical decisions [76]. Users see the advantages of information seeking in OHCs because of the convenience, anonymity, and diversity of information sources.

Specific information is closely related to users’ issues and the content of personal experience [77], such as electronic medical records and online consultations [26]. Moreover, seeking specific information is extremely meaningful for users with similar diseases. For example, specific information is related to specific diagnoses and treatment recommendations, emotional support, and healing experience. Specific information helps users improve health outcomes and makes them perceive benefits. Thus, we propose the following hypotheses.

#### H1

*General information seeking has a positive impact on perceived benefits.*

#### H2

*Specific information seeking has a positive impact on perceived benefits.*

### Effects of general and specific information seeking on perceived costs

The advantage of ICT has made the field of e-health successful [78]. However, seeking and identifying health information in using ICT need to spend on time and energy [8]. OHCs provide substantial content and a variety of sources for information. It is difficult for users to identify the accuracy and reliability of health information. Users find it challenging to seek useful common sense regarding healthcare, particularly when identifying and filtering various claims of unified information. More than 90% of users seek information on their illnesses [14]. Users without e-health literacy seek health information costly.

In addition, specific information has little source credibility, accuracy, currency, and completeness due to user-generated content in OHCs [15]. Users are more likely to incur potential emotional costs, such as sadness and pain, through specific information seeking. Thus, we propose the following hypotheses.

#### H3

*General information seeking has a positive impact on perceived costs.*

#### H4

*Specific information seeking has a positive impact on perceived costs.*

### Effects of perceived benefits and costs on e-satisfaction

The definition of e-satisfaction is “the gratification of people for their previous experience with a certain electronic commerce company” [34]. When the outcomes of general and specific information-seeking match and exceed users’ expectations, they are pleased about seeking experiences. As a form of attitude, satisfaction represents users’ performance in terms of cost, ease of access to resources, and well-being [79]. Prior studies suggest that satisfaction reflects how users’ expectations for products and services are met by comparing them with the expected outcomes [80, 81]. Prior studies have defined satisfaction as an evaluation of health service expectations based on related healthcare requirements [82, 83].

This study proposes that e-satisfaction refers to users’ gratification of general and specific information seeking in OHCs when comparing the expectations with actual experiences. Users receive helpful feedback on their health decisions when they inquire about information and conduct online consultations in OHCs. The consequence of information seeking increases users’ e-satisfaction with OHCs. Users’ perceptions of the advantages of information seeking are invisible and not measurable [24]. Prior studies have found users who are satisfied with the information make for favorable clinical outcomes [84]. However, other issues in OHCs, such as economic costs, misdiagnosis, misleading suggestions, and privacy disclosure, make users perceive costs [85]. Users require significant amounts of time and energy when they use online platforms to seek health information. Costs perceptions of information seeking decrease e-satisfaction with OHCs [86]. Thus, we propose the following hypotheses.

#### H5

*Perceived benefits have a positive impact on e-satisfaction.*

#### H6

*Perceived costs have a negative impact on e-satisfaction.*

## Research design

### Measures

To ensure the validity of the questionnaires, we used well-established scales from prior studies. Items were tested using a 7-point Likert scale (from 1-strongly disagree to 7-strongly agree). As shown in Table 1, scales for general and specific information seeking were adapted from [17] and [24]. Scales for perceived benefits were adapted from [8, 65, 85], and [87]. Scales for perceived costs were adapted from [8] and [88]. E-satisfaction was measured based on [59].

**Table 1:** Measurement items

Constructs	Items	Source
General information seeking (GIS)	GIS1. I typically actively seek general health information when taking part in OHCs	[17, 17]
	GIS2. I frequently participate in the ensuing interactions when discussing matters pertaining to hospitals, medicine, and other public issues	
	GIS3. I frequently spend a lot of time in OHCs looking for general health information	
	GIS4. I frequently participate in general information seeking activities in OHCs	
Specific information seeking (SIS)	SIS1. I frequently participate in the ensuing exchanges when we talk about topics pertaining to treatment-related to issues	[17, 24]
	SIS2. I frequently spend a significant amount of time engaging in specific health information seeking activities in OHCs	
	SIS3. I frequently participate in specific information seeking activities in OHCs	
E-Satisfaction (ES)	ES1. I feel satisfied with the online health information seeking in OHCs	[59]
	ES2. I feel happy with OHCs to search for the health information I need	
	ES3. I feel very content with my seeking health information experiences in OHCs	
Perceived benefits (PEB)	PEB1. I can better manage my health conditions thanks to online health information	[8, 65, 85, 87]
	PEB2. I learn more about my own health conditions when I search for health information online	
	PEB3. Online health resources assist in relieving stress caused by new symptoms or worries about new symptoms	
	PEB4. I find online health information seeking useful in my daily life	
	PEB5. Online health information seeking helps me recover health.{Shiau, 2022 #730}	
	PEB6. Online health information seeking is useful for decision-making	
	PEB7. Online health information seeking helps me solve problems	
	PEB8. Through online health information seeking, I get comfort and care from other patients in OHCs	
Perceived costs (PEC)	PEC1. Health information seeking could be harmful	[8, 88]
	PEC2. My health could be badly impacted by health information seeking	
	PEC3. Because of the subpar quality of online health information, I might decide poorly about my health	
	PEC4. I never seem to have enough time to look for health information in OHCs	
	PEC5. Health information seeking in OHCs is time-consuming	

There were four control variables in our research model, including gender, age, education, and frequency of using OHCs. Since all of the items were crudely developed in English, we translated the English items into Chinese using the reverse translation approach. To ensure the acceptable and reliability of the questionnaires’ content, we invited ten experts to examine whether items were ambiguous. We revised the ambiguous items according to experts’ comments.

### Data collection

We conducted an online survey to verify the previously proposed model. In this study, the subjects of our investigation were Chinese individuals participating in health information seeking in OHCs. The formal survey was conducted on a Chinese online platform. Participants were invited to access the online platform (https://www.wjx.cn/) and complete questionnaires online. We carried out the anonymous online investigation in October 2019. Each participant’s informed permission and privacy were safeguarded before the formal survey.

In addition, we considered participants’ experience of filling online questionnaires to avoid the impact of operation technology. We eliminated from the survey any invalid samples that had no responses and overly quick completed times. The formal survey had 512 participants. 412 valid questionnaires were returned. 75% (412/549) of the data were valid.

### Data analysis

The structural equation model (SEM) was used to examine the hypotheses in our research model. There are two sections in the SEM analysis, including measurement model analysis and structural model analysis. In this study, we adopted PLS-SEM to analyze the hypothesis model and used SmartPLS to analyze the data and test hypotheses.

PLS-SEM estimates biased model relationships in iterative sequences of ordinary least squares regression [88]. PLS-SEM maximizes the explained variance of endogenous latent variables and relaxes the assumption of multivariate normality [89]. PLS-SEM is capable of estimating complex models with small samples [89–91]. The rule of thumb for the sample size is 10–15 times the maximum structural equation [92]. Given the complexity of our research model with five structures and six hypotheses, PLS-SEM is the best approach for this study.

## Results

As shown in Table 2, There were 412 valid responses. Male respondents made up 43.7% of the total, while female respondents made up 56.3% of the total. As for age, 169 respondents were aged from 21 to 30, which covers 41.1% of the total. The number of college respondents is 362 (87.9%). From the perspective of education, respondents with high education levels tend to seek information in OHCs. Moreover, 26 respondents used OHCs almost daily and 170 respondents used OHCs 2–3 times a week on average. 213 respondents used OHCs when health issues appeared, which covers 51.7% of respondents.

**Table 2:** Demographic statistics

Characteristics	Items	Frequency	Percentage (%)
Gender	Male	180	43.7
	Female	232	56.3
Age (years)	20 years and below	8	1.9
	21–30 years	169	41.1
	31–40 years	197	47.8
	41–50 years	33	8.0
	51 years and above	5	1.2
Education	High school (below)	6	1.5
	Junior college	362	87.9
	Master (above)	44	10.7
Frequency of using OHCs	Almost every day	26	6.3
	An average of 2–3 times a week	170	41.3
	Use when needed	213	51.7
	Rarely use	3	0.7

### Measurement model analysis

We used SPSS and SmartPLS to examine the measurement model. Before testing the discriminate validity and convergent validity, we conducted the Kaiser–Meyer–Olkin (KMO) and Bartlett’s test of sphericity. Results indicated that the KMO values were greater than the standard 0.8. Thus, scales are suitable for confirmation factor analysis. Moreover, we used Harmon’s one-factor test to assess common method bias. Results reflected that the first factor accounts for the highest variance of 26.4%. Thus, common method bias was not a severe threat in this study.

As shown in Table 3, the values of average variance extracted (AVE) were over 0.5. The values of composite reliability (CR) were over 0.7. Results reflected the good convergent validity of scales. The values of Cronbach’s alpha were over 0.7. Results indicated the good reliability of scales. As shown in Table 4, correlations between the two constructs were less than the values of the square root of AVE. The discriminate validity of the constructs in this study was identified. The multivariate coefficients of determination results are presented in Table 5.

**Table 3:** Composite reliability and average variance extracted

Constructs	Abb	CR	AVE	C $\mathrm{\alpha }$
General information seeking	GIS	0.891	0.672	0.714
Specific information seeking	SIS	0.866	0.685	0.762
Perceived benefits	PEB	0.973	0.735	0.815
Perceived costs	PEC	0.925	0.712	0.707
E-satisfaction	ES	0.941	0.842	0.741

*CR* Composite reliability, *AVE* Average variance extracted, *C*$\alpha$-Cronbach alpha

**Table 4:** Correlations between constructs

Constructs	Mean	SD	GIS	SIS	PEB	PEC	ES
GIS	5.03	1.29	**0.819**				
SIS	4.32	1.41	0.635	**0.827**			
PEB	5.26	1.15	0.685	0.163	**0.857**		
PEC	2.53	1.21	0.335	0.198	0.039	**0.844**	
ES	5.77	0.973	0.465	0.149	0.533	0.318	**0.917**

The data in bold diagonals refers to square roots of AVE

**Table 5:** Multivariate coefficient of determination (${R}^{2}$) results

Constructs	$\varvec{R}^{\textbf{2}}$		Control variable effects		
	With control variables	Without control variables	$\boldsymbol{\Delta}\varvec{R}^{\textbf{2}}$	$\varvec{f}^{\textbf{2}}$	Effect sizes
PEB	0.699	0.663	0.036	0.119	Weak
PEC	0.243	0.225	0.018	0.024	Weak
ES	0.494	0.461	0.033	0.065	Weak

$$\Delta R^{2} = R_{with\,controal\,variables}^{2} - R_{without\,controal\,variables}^{2}$$; $$f^{2} = Cohen f^{2}$$.

We considered gender, age, education, and frequency of using OHCs as control variables. We used Cohen ƒ^2^ to assess the effects of control variables. Results reflected that the effects of control variables were limited. We evaluated the fitness of the structure model. Table 6 presented several fit indices, including normed chi-square (${\upchi }^{2}/df$), the goodness of fit index (GFI), Tucker-Lewis index (TLI), comparative fit index (CFI), incremental fit index (IFI), root means square error of approximation (RMSEA) and the adjust GFI (AGFI). The values of fit indices reflected the good fitness of our research model.

**Table 6:** Goodness of fit assessments for the structural model

Goodness of fit measures	${\chi }^{2}$(df)	${\chi }^{2}$/df	GFI	IFI	TLI	CFI	AGFI	RMSEA
Goodness of fit ranges		1–3	> 0.9	> 0.9	> 0.9	> 0.9	> 0.9	< 0.05
SEM model	651(406)	1.604	0.911	0.931	0.913	0.929	0.885	0.038

### Structural model analysis

**Fig. 2:**
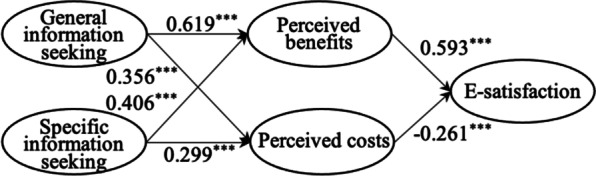
PLS-SEM results of the structural model. *Note*: ***p-value < 0.001

We examined the structural model using PLS-SEM. Table 7 and Fig. 2 present the significance of standard path coefficients. Results indicated that general and specific information seeking positively affected on perceived benefits and costs. Perceived benefits had a positive effect on e-satisfaction. Perceived costs had a negative effect on e-satisfaction.

**Table 7:** Hypothesis testing

Hypothesis	Standard path coefficient	P-value	Result
H1. General information seeking has a positive impact on perceived benefits	0.619	***	Support
H2. Specific information seeking has a positive impact on perceived benefits	0.406	***	Support
H3. General information seeking has a positive impact on perceived costs	0.356	***	Support
H4. Specific information seeking has a positive impact on perceived costs	0.299	***	Support
H5. Perceived benefits have a positive impact on e-satisfaction	0.593	***	Support
H6. Perceived costs have a negative impact on e-satisfaction	− 0.261	***	Support

^***^p-value < 0.001

The results of the effect size analysis are shown in Table 8. General and specific information seeking had significant effects with moderate effect sizes on perceived benefits. General and specific information seeking had effects with weak effect sizes on perceived costs. In addition, perceived benefits and costs had effects with moderate effect sizes on e-satisfaction.

**Table 8:** Partial least squares effect size analysis

Constructs	${R}^{2}$		$\Delta {R}^{2}$	${f}^{2c}$	Effect sizes
	in	out			
*Perceived benefits*					
General information seeking	0.699	0.369	0.330	1.096	Moderate
Specific information seeking	0.699	0.575	0.124	0.412	Moderate
*Perceived costs*					
General information seeking	0.243	0.162	0.081	0.107	Weak
Specific information seeking	0.243	0.194	0.049	0.065	Weak
*E-satisfaction*					
Perceived benefits	0.494	0.349	0.145	0.287	Moderate
Perceived costs	0.494	0.318	0.176	0.348	Moderate

$$\Delta R^{2} = R_{with\,controal\,variables}^{2} - R_{without\,controal\,variables}^{2}$$*;* $$f^{2} = Cohen f^{2}$$*.*

### Robustness check

**Fig. 3:**
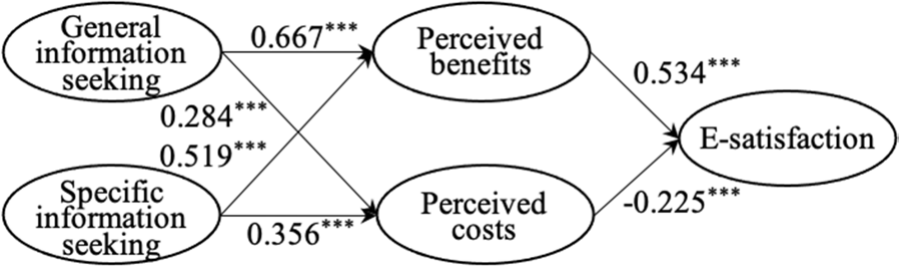
Results of the structural model using AMOS. *Note*: ***p-value < 0.001

To check the robustness of the statistical results, we conducted two steps. First, we used G*Power to calculate whether the 412 sample size has sufficient statistical power. According to post hoc analysis, the power (1 − $\beta$) value above 0.999, exceeding the standard value of 0.8. Thus, our samples could explain the research model. Second, this study measured the structural model using AMOS technique. As shown in Table 9 and Fig. 3, the results of the structural model through using AMOS were consistent with the results of PLS-SEM through using SmartPLS.

**Table 9:** Hypothesis testing

Hypothesis	Standard path coefficient	P-value	Result
H1. General information seeking has a positive impact on perceived benefits	0.667	***	Support
H2. Specific information seeking has a positive impact on perceived benefits	0.519	***	Support
H3. General information seeking has a positive impact on perceived costs	0.284	***	Support
H4. Specific information seeking has a positive impact on perceived costs	0.356	***	Support
H5. Perceived benefits have a positive impact on e-satisfaction	0.534	***	Support
H6. Perceived costs have a negative impact on e-satisfaction	− 0.225	***	Support

^***^p-value < 0.001

## Discussion

We investigate the effects of general and specific information seeking on patients’ e-satisfaction in OHCs. Users in OHCs can perceive benefits and costs through general and specific information seeking. We explore the effects of users’ perceived benefits and costs on e-satisfaction. Perceived benefits make users satisfy the information in OHCs and online platforms.

However, perceived costs can reduce users’ e-satisfaction with OHCs. One of the reasons may be the operation technique of information seeking on the Internet is complicated for users. Participating in OHCs requires users to invest time and effort, which explains why perceived costs lower users’ e-satisfaction. To increase users’ e-satisfaction, perceived benefits in the process of general and specific information seeking should be focused on by OHCs managers.

### Theoretical implications

This study has several theoretical implications. First, this study enriches the literature on users’ different information seeking in OHCs. Information in OHCs is divided into general and specific information [24]. General and specific information seeking make users perceive benefits and costs. According to social exchange theory, perceived benefits have a positive effect on users’ e-satisfaction with OHCs. Conversely, perceived costs negatively affect users’ e-satisfaction with OHCs.

Second, this study extends social information process theory in the context of OHCs. OHCs have two main functions, including information and social supports [93]. After rationally assessing the appropriateness of the information in OHCs, users can perceive benefits and costs. This study contributes to the research about users’ perceived benefits and costs of information seeking in OHCs. Processing general and specific information raises users’ e-satisfaction with OHCs.

Third, this study provides theoretical guidance for increasing users’ e-satisfaction with OHCs. Social information processing theory, as the theoretical framework, explains people’s information behavior and attitude. Previous research has examined users’ satisfaction with online platforms [35]. However, few studies explores the effects of health information seeking on users’ e-satisfaction with OHCs. This study provides an understanding of users’ e-satisfaction from the perspective of general and specific information seeking.

### Practical implications

Meanwhile, this study provides practical implications regarding marketing OHCs. First, this study suggests that OHCs managers should be concerned about users’ perceived benefits of information seeking. This study considers users’ information-seeking as an influential factor in predicting e-satisfaction.

Second, this study suggests that health professionals should focus on health information seeking. The diversity of information and users in OHCs causes users’ perceived costs for information seeking. Results reflect that perceived costs reduce users’ e-satisfaction with OHCs. To increase users’ e-satisfaction, OHCs managers should assist health professionals in seeking information and reduce perceived costs. For example, the user interface of OHCs is easy to operate, reducing users’ time and energy.

Third, OHCs have become an ideal channel for rural patients to access health information [94]. This study investigates information seeking in Chinese OHCs. OHCs are being used by more physicians and patients to access health information [19]. As the world’s largest developing country, the urban–rural health gap in China has always been a serious social problem [95]. This study provides practical implications to increasing users’ e-satisfaction with OHCs for addressing the urban–rural health gap in China.

### Limitations

This study has several limitations and future research directions. First, this study conducts data collection using respondents’ self-reports. The self-rating scales depend on respondents’ knowledge and opinions, which easily causes bias. Future research can use objective data to validate our research model.

Second, cross-sectional data were used in this study, ignoring the time-varying effects of constructs. Future research can use longitudinal data to analyze information seeking in OHCs. Third, this study does not consider the types of OHCs. Different communities may exhibit different ways of online information seeking. Future research can consider different sources of health information.

Finally, the research model of this study does not include the effect of trust in OHCs. Users’ trust is not the main topic of this study. In addition, this study ignores users’ health information needs in OHCs. This is a limitation of our research focusing on e-satisfaction. Further research can explore other issues like trust, misinformation, and information needs in OHCs.

## Conclusion

This study identifies that general and specific information seeking have positive effects on e-satisfaction through the mediation role of perceived benefits. General and specific information seeking affect e-satisfaction negatively by the mediation role of perceived costs. General information seeking has a stronger impact on perceived benefits and costs than specific information seeking. Moreover, perceived benefits have a positive effect on e-satisfaction, while perceived costs have a negative effect on e-satisfaction. Our results suggest that OHCs should increase users’ perceived benefits of general and specific information seeking. Furthermore, OHCs provide general and specific health information for users to improve their e-satisfaction. This study contributes to enriching the literature on the social information process theory in the context of OHCs.

## Data Availability

The authors confirm that the datasets used and/or analysed during the current study available from the corresponding author on reasonable request.
